# And Baalam’s Ass Spake

**Published:** 1901-03

**Authors:** 


### AND BAALAM'S ASS SPAKE.

THERE is a person in Buffalo known among his intimates as Rev. Moses Hull. He is pastor of the Spiritual Temple which more or less flourishes according to the attendance of its habitués. Not knowing where this spiritualistic person received his degree or what claims he has to reverence, we cannot say how well or how illy he has been educated.

However, he preached a sermonette not long ago which is quite the most remarkable bit of brain oozing which has been turned loose in many a day, and it would appear to be worthy of the person who relieved his mind of it. How horrible is the contemplation of what a congregation must suffer at the mouth of such a preacher when they can't talk back. They must just sit and be deluged with rot,—spiritual, temporal, political, social, moral and fashionable,—and say "amen"

with deep grace, and a feeling of earnest thanksgiving that Sunday comes only once in seven days.

But to return to the discourse on vaccination by the Hull person, who talks to spiritualists in a Temple of spiritualism on earthly matters. How bizarre! He started off by jumping into his subject with both feet as it were. After announcing that spiritualism was the advance guard in the interests of humanity, wherein he rather disagrees with his fellow fanatics, the christian scientists, he went on to say, that at the last meeting of the National Association of his faith, the following resolution was passed:

*Resolved,* That compulsory vaccination is not only unwise, unconstitutional and un-American, but dangerous to health, causing eczema, erysipelas, cancers, tumors, and often death.

One might wonder where all this highly interesting information came from, had not the reverend spiritualist in the next paragraph said this:

Tuberculosis should have come in among the evils here enumerated, for vaccination is the cause of more pulmonary consumption than any other thing in the world. I was chairman of this committee on resolutions, and I have ever regretted my shortsightedness in omitting that one word.

He should have gone on and not content with adding tuberculosis, hitched on a few other ailments. Of course, he overlooked piles, and pus, and pregnancy, but maybe he will take them up at some future time, provided he can reconcile those conditions with spiritualism.

Facts from every part of the world, he continued, will demonstrate that at least one half of the consumption originates in vaccination. If the charges in this resolution are true, vaccination not only fees a gang of hungry doctors at the expense of the public, but it lays the foundation for future harvests at the expense of a suffering public.

We affirm in this resolution that vaccination is unwise. It teaches a bad lesson; it takes the mind from the true remedy for smallpox, and it has caused the public, which trusts in its doctors, so-called, to cease to look for the true cause and real remedy for smallpox.

I now assert that it is unconstitutional and un-American for a policeman to come in with a club, and assist a butcher with a lancet in arresting our children, who have committed no crime—to overcome them by the power of brute force, and inject into their system a poison worse than any rattlesnake carries in his fangs. Such things as vaccination of terrible criminals for heinous offenses, might do to go with capital punishment. Under any other circumstances, it is too barbarous to be tolerated in an enlightened republic.

Nor is it just to deprive children of an education, because their parents, their natural protectors, refuse to have them poisoned. These laws have been enacted in nearly all of the states. It is the worst kind of class legislation, a legislation which, in New York City alone, will this year put $4,000,000 of the people's money into the pockets of the members of one profession, and at the same time lay the foundation for disease enough to keep the doctors busy all their lives. It is the worst kind of class legislation, and has been so decided. I would prefer to give the doctor a thousand dollars to keep away from my house, than to pay him one dollar to come in and poison my family.

And so he discoursed, preaching the gospel on Sunday, damning the doctors, and talking rot.

The BUFFALO MEDICAL JOURNAL is a firm believer in religious freedom. Every man has an inalienable right to worship as he pleases, how he pleases and at his own convenience, be he spiritualist, christian scientist, or Dowieite. Christianity and education draw the line sharply at idolatry and fanaticism, and that's where these classes of worshipers at fad shrines come in. The christian scientists idolise mother Eddy; and Mr. Hull, to be charitable, is fanatic. A grown up christian scientist has a perfect right to decline medical attendance, if that's his honest belief, but he has no legal or moral right to deprive his baby of proper attention when it is ill, no more than the Hull person has to use his pulpit and position to disseminate such blatant error. Thinking over this eminent spiritualistic divine's discourse, one cannot help coming to the conclusion that after all smallpox isn't the worst thing in the world.

